# Polar lipids modify Alzheimer’s Disease pathology by reducing astrocyte pro-inflammatory signaling through platelet-activating factor receptor (PTAFR) modulation

**DOI:** 10.1186/s12944-024-02106-z

**Published:** 2024-04-20

**Authors:** Sakshi Hans, Janelle E. Stanton, Ann Katrin Sauer, Katie Shiels, Sushanta Kumar Saha, Ronan Lordan, Alexandros Tsoupras, Ioannis Zabetakis, Andreas M. Grabrucker

**Affiliations:** 1 Department of Biological Sciences, University of Limerick, Limerick, V94PH61 Ireland; 2 Bernal Institute, University of Limerick, Analog Devices Building AD3-018, Castletroy, Limerick, V94PH61 Ireland; 3 Health Research Institute (HRI), University of Limerick, Limerick, V94PH61 Ireland; 4 Shannon Applied Biotechnology Centre, Technological University of the Shannon, Moylish Park, Limerick, V94E8YF Ireland; 5 Institute for Translational Medicine and Therapeutics, Perelman School of Medicine, University of Pennsylvania, Philadelphia, PA USA; 6 Department of Medicine, Perelman School of Medicine, University of Pennsylvania, Philadelphia, PA USA; 7 Department of Systems Pharmacology and Therapeutics, Perelman School of Medicine, University of Pennsylvania, Philadelphia, PA USA; 8 Hephaestus Laboratory, Department of Chemistry, School of Science, Democritus University of Thrace, Kavala University Campus, Kavala, GR65404 Greece

**Keywords:** Polar lipids, Alzheimer's Disease, Beta-amyloid, Neuroinflammation, Nutrition, Astrocytes, Glial cells

## Abstract

**Background:**

Pro-inflammatory processes triggered by the accumulation of extracellular amyloid beta (Aβ) peptides are a well-described pathology in Alzheimer's disease (AD). Activated astrocytes surrounding Aβ plaques contribute to inflammation by secreting proinflammatory factors. While astrocytes may phagocytize Aβ and contribute to Aβ clearance, reactive astrocytes may also increase Aβ production. Therefore, identifying factors that can attenuate astrocyte activation and neuroinflammation and how these factors influence pro-inflammatory pathways is important for developing therapeutic and preventive strategies in AD. Here, we identify the platelet-activating factor receptor (PTAFR) pathway as a key mediator of astrocyte activation. Intriguingly, several polar lipids (PLs) have exhibited anti-inflammatory protective properties outside the central nervous system through their inhibitory effect on the PTAFR pathway. Thus, we additionally investigated whether different PLs also exert inhibitory effects on the PAF pathway in astrocytes and whether their presence influences astrocytic pro-inflammatory signaling and known AD pathologies in vitro.

**Methods:**

PLs from salmon and yogurt were extracted using novel food-grade techniques and their fatty acid profile was determined using LC/MS. The effect of PLs on parameters such as astrocyte activation and generation of oxygen species (ROS) was assessed. Additionally, effects of the secretome of astrocytes treated with these polar lipids on aged neurons was measured.

**Results:**

We show that PLs obtained from salmon and yogurt lower astrocyte activation, the generation of reactive oxygen species (ROS), and extracellular Aβ accumulation. Cell health of neurons exposed to the secretome of astrocytes treated with salmon-derived PLs and Aβ was less affected than those treated with astrocytes exposed to Aβ only.

**Conclusion:**

Our results highlight a novel underlying mechanism, why consuming PL-rich foods such as fish and dairy may reduce the risk of developing dementia and associated disorders.

**Supplementary Information:**

The online version contains supplementary material available at 10.1186/s12944-024-02106-z.

## Background

Increased concentration and aggregation of the amyloid beta peptide (Aβ) and tau hyperphosphorylation resulting in neurofibrillary tangles are hallmarks of Alzheimer's disease (AD) pathology. Associated with these features are impaired synaptic function, neuroinflammation, neurodegeneration, and the resulting cognitive and behavioral deficits. Recent research indicates that especially pro-inflammatory processes play a crucial role as drivers of AD progression [1, 2]. Besides microglia, astrocytes seem to be key mediators of the inflammatory response in AD [3]. Activation of astrocytes is hypothesized to occur via the Receptor for Advanced Glycation Endproducts (RAGE; gene name *AGER*) receptors binding Aβ and leads to further activation of downstream signaling pathways such as p38 MAPK and nuclear factor kappa B (NF*-*κB) [4, 5]. When AGE-like ligands or Aβ bind RAGE, phosphorylation of p38 MAPK occurs that activates the transcription factor NF-κB. NF-κB then promotes the transcription and expression of pro-inflammatory cytokines such as interleukin-1β (IL-1β), tumor necrosis factor-α (TNF-α), interleukin-6 (IL-6), and Cyclooxygenase-2 (COX2) [5–7]. Aβ-RAGE interaction also regulates the protein kinase R-like endoplasmic reticulum kinase (PERK) pathway. The PERK protein phosphorylates the eukaryotic translation initiation factor 2α (eIF2α) protein kinase. Phosphorylated eIF2α induces the expression of activating transcription factor 4 (ATF4), which in turn promotes the expression of unfolded protein response (UPR), resulting in apoptosis [7, 8]. Thus, Aβ-RAGE interaction promotes the activation of multiple signaling pathways in astrocytes and the expression of proteins that encourage a pro-inflammatory state.

These reactive astrocytes have been shown to affect synapse function and neuronal health in AD [9, 10]. Although the underlying mechanisms are currently not fully understood, signaling through signal transducer and activator of transcription 3 (STAT3) and/or NF*-*κB likely mediates these effects [11–13]. In particular, the NF*-*κB activation-dependent release of pro-inflammatory cytokines IL-1β and IL-6 from astrocytes [14] may be a key inflammatory stimulus in AD. Intriguingly, anti-inflammatory therapies targeting astrocytes have shown promising effects in AD models [3]. However, it is currently less known which ligands and receptors activate the downstream signaling pathways.

One known activator of NF*-*κB signaling outside the brain is the platelet-activating factor receptor (PTAFR) pathway. Its activation produces pro-inflammatory cytokines and mediators via activation of NF-κB signaling and other cellular signaling pathways [15]. High platelet-activating factor (PAF) (1-*O*-alkyl-2-acetyl-*sn*-glycero-3-phosphocholine) levels may promote inflammation as well as drive many inflammation-related diseases, including allergies and viral infections [16]. It is a key signaling molecule in the renal, cardiovascular, immune, and reproductive systems [17]. Initially, as the name suggests, PAF was described as a platelet activator via binding to the PTAFR, which ultimately induces platelet aggregation [18] However, the PTAFR is expressed in many cells of organs, such as in the lungs, spleen, heart, kidneys, skeletal muscle, and, notably, in the brain [17]. PAF is synthesized by neuronal tissue [19]. Thus, it has been speculated that PAF may also play a role in cell signaling in the central nervous system (CNS) by activating the NF-κB pathways [16, 20]. Indeed, the PTAFR is highly expressed in microglia and PAF-induced neuron inflammatory responses in *in vitro* experiments [21]. In line with previous research, we report that PTAFR is also expressed in astrocytes [22].

Interestingly, it has been shown that polar lipids (PLs), phospholipids and sphingolipids, can act as PAF analogs and significantly reduce or prevent PTAFR activation through competition with PAF for the PTAFR or via modulation of PAF metabolism [17]. Lipids are a very diverse group of molecules divided into two major classes: neutral lipids and polar lipids (PLs) (such as phospholipids and glycolipids). Recently, PLs have been highlighted as important inhibitors of the inflammatory response outside of CNS, where PLs were shown to have anti-inflammatory and antithrombotic effects. These effects were reached by influencing PAF-dependent pathways [23]. PAF is released in the early stages of inflammation [24]. For example, the structure of some marine PLs (e.g., extracted from salmon tissue) share similarity to the structure of PAF, and, therefore, these PLs can block the PTAFR. However, whether PLs exert this activity in the presence of Aβ-induced astrocyte activation and AD pathology is currently unknown.

The brain mainly utilizes acylated lipids to generate phospholipids for cell membranes [25] Although the prominent role of PLs is to support the formation and bio-functionality of cell membranes, specific PLs may also act as mediators of inflammatory responses in the brain. Marine phospholipids have been shown to possess many nutritional advantages, including the fact that they increase the bioavailability of polyunsaturated fatty acids (PUFAs), specifically n-3 fatty acids, such as eicosapentaenoic acid (EPA) and docosahexaenoic acid (DHA). In addition, recent studies have shown that DHA can help boost the clearance of Aβ in the plasma and aid in preventing AD [26] and PL ingestion has been linked to cognitive function [27]. However, we speculate that the beneficial effects of PLs may also be realized through their ability to interfere with PAF signaling in the brain, which ultimately will attenuate astrocyte activation and the associated cytokine release. Therefore, here, we investigated whether inflammatory responses of astrocytes triggered by the presence of Aβ can be influenced by exposure to PLs in a PTAFR-dependent mechanism. We evaluate whether PL-mediated astrocyte modulation is sufficient to alter AD pathology *in vitro*. Further, we explore PLs from different sources to evaluate whether a specific PL profile is more potent in inhibiting the PTFAR pathway.

## Materials and methods

### Materials

DI TNC1 (ATCC® CRL-2005™) rat astrocyte cells were used as the cell culture model. Lipid extraction reagents (hexane, ethanol, and water) were obtained from Fisher Scientific (Ireland). Poly-L-lysine (PLL), Dulbecco's modified eagle medium (DMEM), dimethyl sulfoxide (DMSO), sodium pyruvate, trypsin, and penicillin-streptomycin were obtained from Sigma-Aldrich (Ireland). Hank's balanced salt solution (HBSS), Fetal bovine serum (FBS), GlutaMAX (glutamate), and phosphate-buffered saline (PBS) were obtained from Gibco (BioScience, Ireland). Human Beta-Amyloid peptide 1-42 (Aβ) was obtained from Abcam (ab120301). The SYBR Green PCR and RNeasy kit were sourced from Qiagen (UK). Unless otherwise indicated, all chemicals were obtained from Sigma-Aldrich/Merck (Ireland). CellROX Green Dye was obtained from Thermofisher. Primary antibodies were purchased from Biolegend (GFAP, cat. no. 644702: ICC: 1:250, WB: 1:200; β-tubulin, cat. no. 607152: WB: 4 µg/mL; MAP2, cat. no. 840601, ICC: 1:1000), Abcam (PAFR, cat. no. ab104162: ICC: 1:25, WB: 1:200), ProteinTech (β-actin, cat. no. 20536-1 AP: WB 1:1000), and Synaptic Systems (Shank3, cat. no. 162 304: ICC: 1000). Secondary AlexaFluor antibodies were purchased from Invitrogen and HRP-conjugated antibodies for WB were sourced from Dianova and Dako. Unless otherwise indicated, all other chemicals were obtained from Sigma-Aldrich (Merck, Ireland). The Annexin V staining kit was purchased from Leinco Technologies (US).

### Polar lipid preparation

Samples of Irish salmon fillet and Irish ovine yogurt were obtained. A food-grade salmon extract and ovine yogurt polar lipid sample was prepared as per Tsoupras et al. [28]. The yogurt was prepared similarly as in Lordan et al. [29], with the fermentation performed at 40.5°C for 7h until the yogurt reached a pH of 4.6. Briefly, samples (*n* = 3) of fresh salmon fillets and ovine yogurt (100 gm) were homogenized mechanically in a Waring blender, and from these, the total lipid content (TL) was extracted. From the TL fraction, the neutral lipids (NL) and polar lipid (FGE-PLs) fractions were further isolated using food-grade solvents, namely water, ethanol, and hexane (Fisher Scientific), according to EU legislation for food-grade based extractions of fish oil (consolidated Directive 2009/32/EC: https://eur-lex.europa.eu/legal-content/en/ALL/?uri=CELEX:32009L0032). Solvents were evaporated from the samples using flash rotary evaporation (Buchi Rotavapor, Mason Technology), and lipid samples were transferred to small glass vials, where all the remaining solvents were further evaporated under a stream of nitrogen. Excess solvent was evaporated under a steady stream of nitrogen from the polar lipid extracts thus obtained. Samples were stored at -20℃ until further use.

### Cell culture

Astrocytes: (Rat DI TNC1 cells) were cultured in DMEM High Glucose medium supplemented with 10% FBS, 2% glutamate, 1% streptomycin/penicillin, and 1% sodium pyruvate and seeded on cell culture dishes coated with PLL until they reached circa 80% confluency for use in experiments. All cell lines were maintained in a humidified incubator at 37°C and 5% CO_2_.

Primary Neurons: Rat primary hippocampal neurons from day 18 embryonic rats (Innoprot) were plated at a density of 52,000 cells per coverslip in 24-well cell culture dishes coated with PLL. The neurons were grown in Neurobasal A Medium (Gibco) supplemented with 1% Glutamax, 1% streptomycin/penicillin and 2% B-27. After plating on DIV0, the neurons were cultured at 37 °C and 5% CO_2_ for 14 days.

### Treatment of cells

A stock solution of 200 μM of human Aβ_1-42_ was prepared in DMSO. After vortexing the stock solution for 30 min at room temperature (RT), the solution was centrifuged (DLAB D3024R) for 1 h at 4 °C at 15,000 x g. Resuspension of lyophilized Aβ_1-42_ usng this method should result in mostly monomeric Aβ, but may not completely remove pre-aggregates. However, all treatments have been performed using the same Aβ preparation, and cells in all conditions were exposed to Aβ in the same initial aggregation state. For treatments, cells were grown to circa 70% confluency on Petri dishes and treated with a final concentration of 1 µM Aβ peptide for 24 h. Control groups were cultivated without peptides simultaneously. Treatment with polar lipids: Stock solutions of salmon fillet polar lipid (SF-Pls) extract and ovine yogurt polar lipids (YPL) were made in 0.9% NaCl/0.05% mg/ml BSA buffer in a 100 mg/ml concentration. For treatments, a working concentration of 150 µg/ml of PLs diluted in media was used, and cells were incubated for 24 h. To eastblish this concentration, we exposed astrocytes to different concentrations (100, 150, 400 and 800 µg/ml) of PLs. Our results showed that 150 µg/ml has no effect on cell health or proliferation over 24 hours, measured by impedance using an xCelligence RTCA instrument, while higher concentrations reduced cell proliferation/health (data not shown). After 24 h, old media was discarded, and new media was added to the cells. For LPS treatment, a final concentration of 1 µg/µl LPS (lipopolysaccharides from *Escherichia coli* O127:B8, Sigma Aldrich) was used. Cells were incubated for 24 h at 37 °C and 5% CO_2_.

Neurons on DIV 11 were treated with media collected from astrocytes treated as described above, in a ratio of 60:40 (neuronal complete media: astrocyte complete media). The neurons thus treated were left in this ratio of media until DIV 15, after which they were stained to assess cell health and synaptic density, as described in sections below.

### Gene expression analysis

The total RNA was isolated using the RNeasy Mini kit (Qiagen, Manchester, UK) according to the manufacturer's instructions. First-strand synthesis and real-time qRT-PCR amplification (Roche LightCycler 480 II) were carried out in a one-step, single-tube format using the Rotor-Gene SYBR Green RT-PCR kit (Qiagen) and using validated primer pairs from Qiagen (Quantitect primer assay). The hydroxymethylbilane synthase (HMBS) gene was used as an internal standard. All reactions were run at least in technical triplicates, and mean ct values were transformed into virtual mRNA levels according to the formula: virtual mRNA level = 10 × ((ct(target) – ct(standard))/slope of the standard curve).

### Protein expression analysis

Protein extraction for Western blot was carried out by the addition of lysis buffer A (0.5 M HEPES and 1 M sucrose, pH 7.5) containing one dissolved cOmplete mini EDTA-free protease inhibitor cocktail tablet (Roche, Merck) to cells on ice for 15 minutes. The cell lysate was centrifuged at 3,200 rpm (963 x g) for 15 minutes at 4 °C to isolate proteins. The resulting supernatant was collected and stored at -20 °C until further use in SDS-PAGE. The protein concentration was measured using the Bradford assay, and 30 µg of protein was loaded into SDS-PAGE gels with 4X SDS sample-loading buffer.

Proteins were separated by SDS–PAGE and blotted onto nitrocellulose membranes. The blots were probed with primary antibody at 4 °C overnight. Following this, the blots were visualized using HRP-conjugated secondary antibodies and Pierce™ ECL Western Blotting Substrate kit (Thermo Fisher Scientific).Western blot membranes were then imaged using Alliance Q9 Advanced equipment and software from UVITEC. Evaluation of protein band intensity from WBs was performed using ImageJ.

### Immunocytochemistry

Cells grown on glass coverslips were washed with 1X PBS and fixed with 4% PFA for 15 min at room temperature (RT). After removing the PFA, the cells were washed with 1X PBS thrice for 5 min each at RT. The cells were then blocked with blocking solution (BS) (10% FBS/1X PBS) for 1 h at RT to avoid unspecific binding of antibodies. Following this, cells were incubated with the primary antibody dissolved in BS overnight at 4 °C, followed by three washing steps with 1X PBS to wash off any unbound primary antibody. The secondary antibody was diluted in BS and incubated for 1 h at RT. Following this, the cells were washed with 1X PBS thrice for 5 min each, and the cell nuclei were counterstained with DAPI. A final step was performed in which cells were washed with distilled water to remove salt crystals. Finally, the cells were mounted onto glass slides with Vectamount aqueous mounting medium.

### Assessment of oxidative stress

Cells were stained with CellROX Green Dye (Thermofisher) to assess intracellular oxidative stress. Astrocytes were grown on a PLL-coated Greiner multiwell dish until circa 70% confluency and then live staining with CellROX Green (5 µM) for 30 min at 5% CO_2_ and 37 °C. In the oxidative stress positive control, astrocytes were treated with CuCl_2_ (2 μM) associated with H_2_O_2_ (10 μM) for 3 hours to induce a Fenton reaction before staining with CellROX Green. Following this, cells were washed with 1X PBS and fixed with 4% PFA for 15 min at RT. Cell nuclei were counterstained with DAPI, and cells were left in 1X PBS for imaging. Cellular imaging was performed using the ImageXpress Micro Confocal (Molecular Devices), and images thus acquired were analyzed with ImageJ.

### Assessment of cell health

Assessment of cell health or live-dead cell staining was performed using Annexin V-FITC. Rat hippocampal neurons were washed twice with 1X binding buffer containing calcium chloride before staining. The staining solution was prepared in 1X binding buffer containing Annexin V, propidium iodide (red), and Hoechst 3342, and neurons were incubated for 15 min in the dark at RT. After this, neurons were washed twice with 1X binding buffer and fixed in 4% PFA with calcium chloride. After removing the PFA, neurons were mounted on glass slides with Vectamount mounting medium.

### Statistics

Lipid extractions were completed in triplicates and the identified fatty acid values are averages of triplicates. Statistical analysis was performed using Graph Pad Prism Version 8.4.2 (464) (La Jolla, CA, USA), and values were tested for significance using one-way ANOVA followed by Tukey post hoc tests or t-tests for pair-wise comparison. All values were normally distributed. Statistical tests were two-tailed with a significance level of α ≤ 0.05. Significances are stated with *p* values <0.05*; <0.01**; <0.001***. Results are shown as mean and SEM.

## Results

**Fig. 1:**
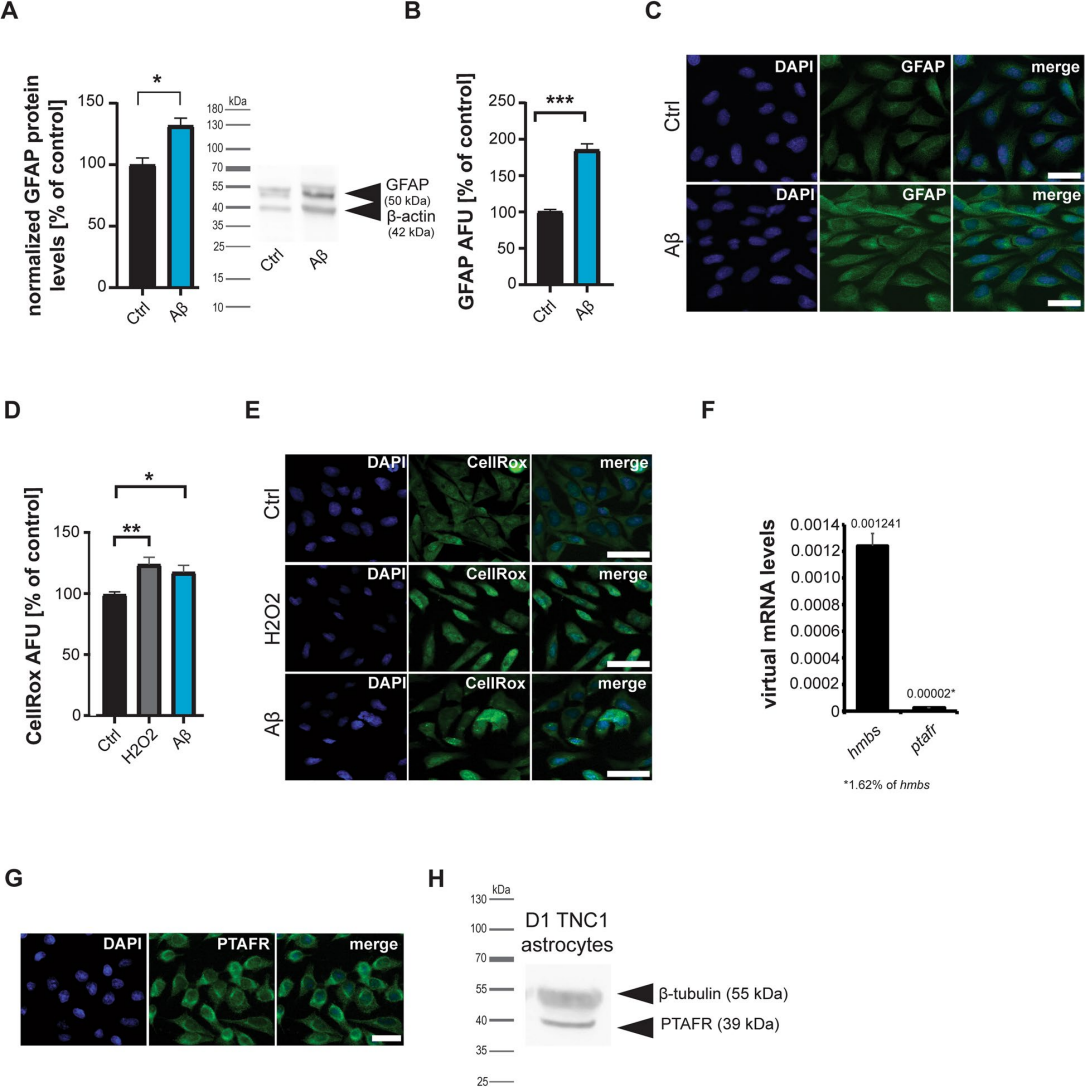
**A-E** DI TNC1 cells were treated with 1 µM Aβ peptide for 24 h and compared to untreated and positive controls. **A** Western blotting (WB) reveals an increase of GFAP on protein level after exposure to Aβ (*t*-test, *p*=0.0183, *n*=3). **B**, **C** Immunocytochemistry (ICC) labeling of GFAP after exposure to Aβ confirms an increase seen by WB (*t*-test, *p*<0.0001, *n*=15) (AFU: Absolute Fluorescence Units). **C** Exemplary images of GFAP signals after treatment. **D**, **E** CellRox^®^ immunoreactivity significantly increases after Aβ treatment, indicating higher ROS levels. H_2_O_2_ was used as a positive control to confirm the sensitivity of the fluorescent marker. As expected, adding H_2_O_2_ also significantly increases CellRox^®^ fluorescence intensity (one-way ANOVA, *p*=0.0068; Tukey posthoc test: Ctrl vs. H_2_O_2_ *p*=0.0067; Ctrl vs. Aβ *p*=0.044, *n*=10). **E** Exemplary images of CellRox^®^ fluorescence after treatment. **F-H** Detection of PTFAR in astrocytes on mRNA level using qRT-PCR (**F**) and protein levels using ICC (**G**) and WB (**H**). **F** *ptafr* mRNA was detected at a level of 1.62% of the housekeeping gene *hmbs* in DI TNC1 astrocytes. **G** Anti-PTAFR antibodies show positive labeling in DI TNC1 astrocytes. **H** The same anti-PTAFR antibodies used in ICC (G) reveal a protein band at the expected size for PTAFR in WB. **C**, **E**, **G** Scale bar = 30 μm

In the first set of experiments, we investigated whether the presence of Aβ results in an activation of astrocytes. To that end, astrocytes were treated with 1 µM Aβ for 24 hours. Astrocyte activation was measured through the assessment of glial fibrillary acidic protein (GFAP) levels (Fig. 1A-C), a known marker for astrocyte reactivity, and the levels of reactive oxygen species (ROS) (Fig. 1D, E), given that activated astrocytes are generating higher levels of ROS [30]. Aβ significantly increased GFAP levels assessed by Western blotting (Fig. 1A) and quantitative fluorescent microscopy (Fig. 1B, C). There was no significant difference in activation between untreated controls and vehicle controls (Supplementary Fig. S1), and untreated controls are shown in the figures in the main text. Similarly, Aβ significantly increased astrocytic ROS levels assessed by quantitative fluorescent microscopy using a dye reacting to ROS (CellROx®)(Fig. 1D, E). As a positive control, cells were treated with H_2_O_2_. H_2_O_2_ interacts with transition metal ions such as copper and iron in cells and medium in the Fenton reaction, generating ROS.

Next, we investigated whether PTAFR is expressed in astrocytes and whether its signaling contributes to their observed activation. We could detect PTAFR using gene expression analysis with *ptafr* mRNA-specific primers (Fig. 1F), immunocytochemistry (Fig. 1G), and protein biochemistry (Fig. 1H).

**Fig. 2:**
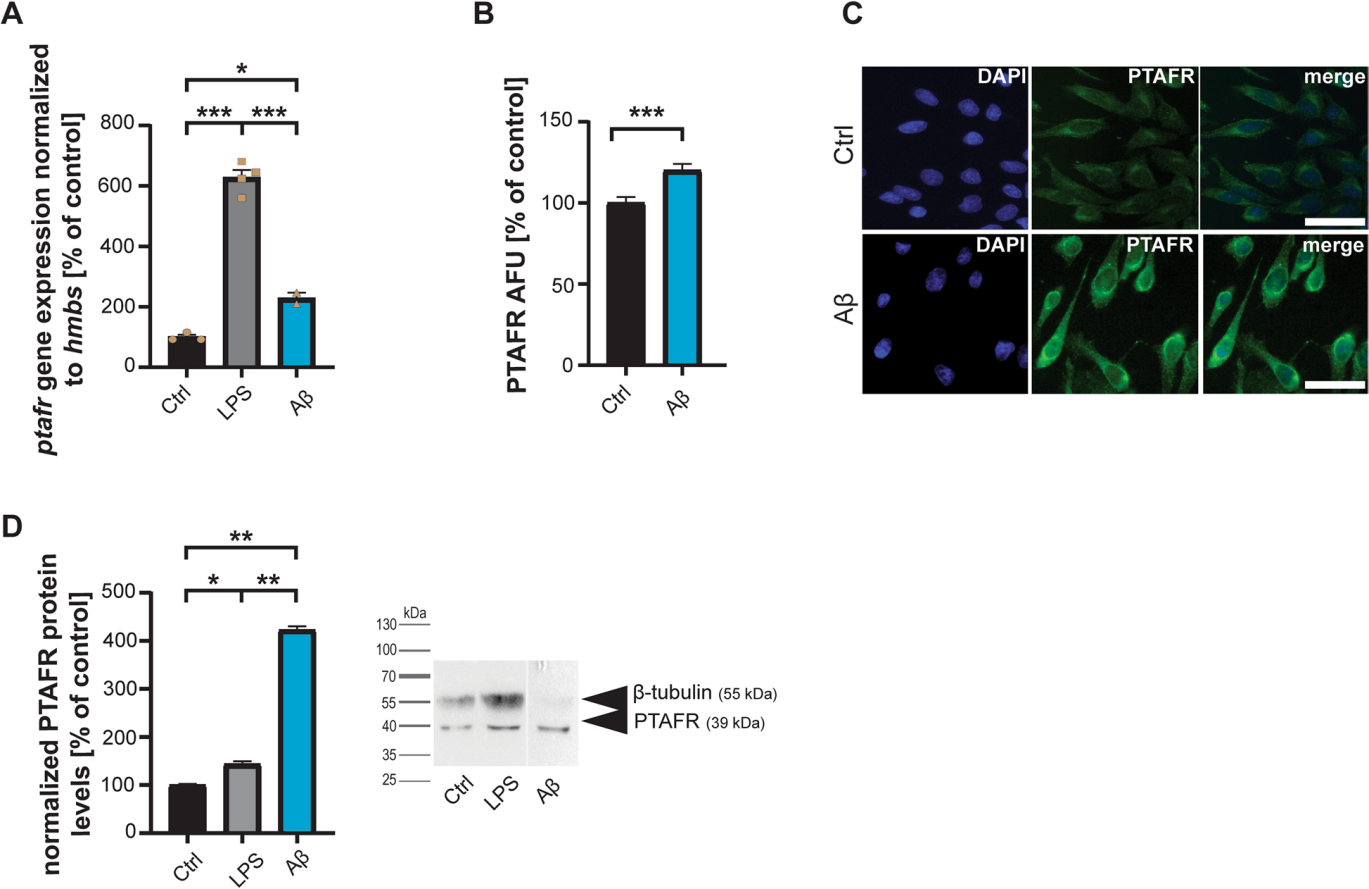
**A** DI TNC1 cells were treated with 1 µM Aβ peptide or 1 µg/µl LPS for 24 h and compared to untreated controls. qRT-PCR using rat *ptafr* specific primers and *hmbs* as housekeeping gene for normalization reveals a significant increase in *ptafr* gene expression after activation of astrocytes by LPS and Aβ (one-way ANOVA, *p*<0.0001; Tukey posthoc test: Ctrl vs. LPS *p*<0.001; Ctrl vs. Aβ *p*=0.0235; Aβ vs. LPS *p*<0.001, *n*=3-4). **B**, **C** Immunocytochemistry confirms the increase in protein level. 24 h after treatment with 1 µM Aβ peptide, PTAFR fluorescent signals are significantly increased (*t*-test, *p*=0.0008, *n*=15). (AFU: Absolute Fluorescence Units). **C** Exemplary images of PTAFR fluorescence after treatment. Scale bar = 30 μm. **D** Western blotting further confirms an increase of PTAFR on protein level after exposure to LPS and Aβ (one-way ANOVA, *p*<0.0001; Tukey posthoc test: Ctrl vs. LPS *p*=0.0145; Ctrl vs. Aβ *p*=0.0100; Aβ vs. LPS *p*=0.00100, *n*=3). Tubulin was used as a housekeeping protein for normalization. The exposure time was reduced for the Aβ condition to avoid overexposure of the PTAFR band

LPS treatment increased the expression of PTAFR, hinting at the role in pro-inflammatory responses of astrocytes (Fig. 2A). Notably, adding Aβ also significantly increased PTAFR expression detected on mRNA level (Fig. 2A) and protein level (Fig. 2B-D). A significant increase in PTAFR immunofluorescence was observed after Aβ mediated astrocyte activation (Fig. 2B,C). This increase was confirmed by protein biochemistry (Western blotting) (Fig. 2D).

**Fig. 3:**
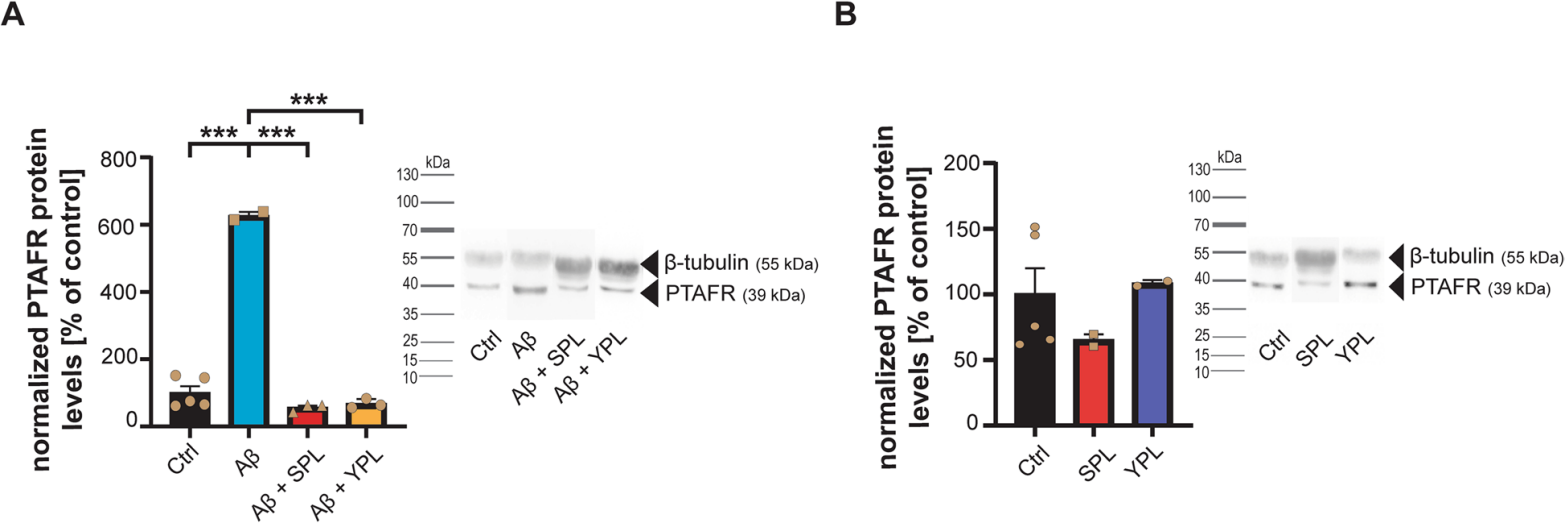
**A** Western blotting reveals an increase of PTAFR on protein level after exposure to Aβ that is normalized by treatment with SPL and YPL (one-way ANOVA, *p*<0.0001; Tukey post hoc test: Ctrl vs. Aβ *p*<0.0001; Aβ vs. Aβ+SPL *p*<0.001; Aβ vs. Aβ+YPL *p*<0.001, *n*=3-5). **B** Western blotting shows no effect of SPL and YPL alone on PTAFR levels (one-way ANOVA, *p*=0.471, *n*=3-5). **B**, **C** Tubulin was used as a housekeeping protein for normalization

Thus, next, we evaluated whether specific inhibition of PTAFR by PLs can reduce PTAFR expression and, thereby, potentially reduce or even prevent astrocyte activation. Therefore, we treated cells again with Aβ and measured PTAFR, GFAP, and ROS levels. However, we included PLs from two different sources (Fig. 3A): salmon-derived PLs (SPL) and yogurt-derived PLs (YPL). The fatty acid profiles of SPL and YPL are shown in Table 1. Astrocytes were treated with 150 µg/ml of PLs for 24 h to saturate the PTAFR with PLs. Then, 24 h treatment with Aβ was performed as before. Our results show that a significant reduction of the increased PTAFR protein expression induced by Aβ can be observed for SPL and YPL using Western blotting (Fig. 3A). Treatment of cells with SPL and YPL alone, in the absence of Aβ, showed no effects on PTAFR levels (Fig. 3B).

**Table 1:** Fatty acid profile of salmon (SPL) and yogurt polar lipid (YPL) extract. Results are expressed as a percentage of each fatty acid of the total fatty acid content of each sample (*n* = 3)

**Fatty Acid Name**	**Mass**	[M-H]^-^	**SPL**		**YPL**	
			**mean**	**SD**	**mean**	**SD**
Caprylic	144.115	143.108	ND	ND	6.62	0.13
Pelargonic	158.131	157.123	0.00	0.00	1.33	0.10
Capric	172.146	171.139	0.00	0.00	7.69	1.65
Undecylic	186.162	185.155	ND	ND	1.92	0.22
Lauric	200.178	199.170	ND	ND	3.63	0.31
Tridecylic	214.193	213.186	ND	ND	2.23	0.22
Myristic	228.209	227.202	3.22	0.17	2.97	0.05
Pentadecylic	242.225	241.217	0.15	0.01	6.41	0.45
Palmitic	256.240	255.233	17.01	0.62	13.95	0.80
Palmitoleic	254.225	253.217	3.92	0.09	4.16	0.12
Margaric	270.256	269.249	0.72	0.09	13.58	0.61
Stearic	284.272	283.264	2.16	0.17	1.83	0.05
Oleic	282.256	281.249	11.41	0.05	7.60	0.30
Linoleic	280.241	279.233	42.19	0.67	3.22	0.29
Linolenic (α + γ)	278.225	277.217	2.50	0.13	3.00	0.13
Stearidonic	276.209	275.202	0.26	0.01	0.23	0.01
Nonadecylic	298.287	297.280	0.00	0.00	0.10	0.01
Arachidic	312.303	311.296	ND	ND	4.33	1.26
Gadoleic	310.287	309.280	2.70	0.21	7.96	0.51
DihomoLinoleic	308.272	307.264	1.69	0.27	1.96	0.18
Dihomolinolenic	306.256	305.249	1.27	0.03	1.70	0.18
Arachidonic	304.240	303.233	1.95	0.08	1.42	0.14
EPA	302.247	301.217	2.51	0.32	0.19	0.02
Docosadienoic	336.303	335.296	0.03	0.00	ND	ND
Eranthic	334.287	333.280	0.01	0.00	0.34	0.03
Ardenic	332.272	331.264	0.26	0.01	0.57	0.06
DPA	330.256	329.249	2.19	0.16	0.91	0.09
DHA	328.240	327.233	3.82	0.33	0.14	0.01
**SFA**			**23.27**	0.81	**66.60**	1.17
**MUFA (n-9)**			**18.00**	0.09	**19.73**	0.34
**PUFA**			**58.40**	0.92	**12.77**	0.77
**n-3 (PUFA)**			**11.30**	0.60	**4.48**	0.12
**n-6 (PUFA)**			**47.10**	0.37	**8.30**	0.72
**n-6/n-3**			**4.17**	0.25	**1.85**	0.21

*Abbreviations*: *SFA* Saturated fatty acids, *MUFA* Monounsaturated fatty acids, *PUFA* Polyunsaturated fatty acids, *n-6* omega-6, *n-3* omega-3, *DHA* Docosahexaenoic acid, *EPA* Eicosapentaenoic acid, *DPA* Docosapentaenoic acid; and *ND* Non-detectable

**Fig. 4:**
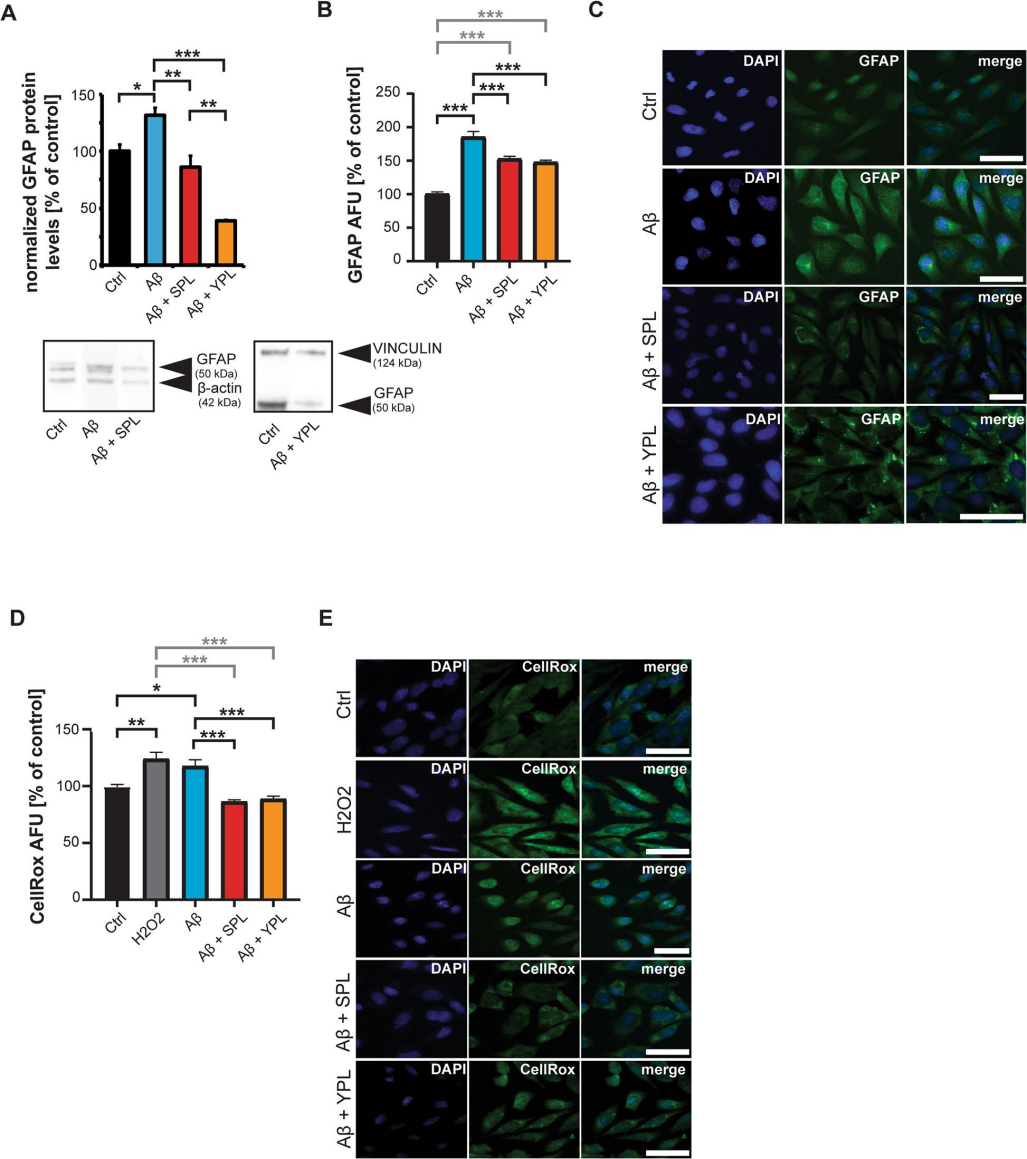
**A-E** DI TNC1 cells were treated with 1 µM Aβ peptide for 24 h, and Aβ with SPL and YPL, and compared to untreated controls and positive controls. **A** Western blotting (WB) reveals an increase of GFAP on protein levels normalized to β-ACTIN (Ctrl, Aβ, and Aβ+SPL) or VINCULIN (Ctrl, and Aβ+YPL) after exposure to Aβ. The results are shown as % of respective Ctrl. The increase is normalized by SPL and YPL treatment (one-way ANOVA, *p*<0.0001; Tukey post-hoc test: Ctrl vs. Aβ *p*=0.0173; Aβ vs. Aβ+SPL *p*=0.0046; Aβ vs. Aβ+YPL *p*<0.001; Ctrl vs. Aβ+YPL *p*=0.0004; Aβ+SPL vs. Aβ+YPL *p*=0.0038, *n*=3). **B**, **C** Immunocytochemistry (ICC) labeling of GFAP after exposure to Aβ confirms the effects of SPL and YPL. The increase in GFAP induced by Aβ is significantly reduced in SPL and YPL-treated astrocytes (one-way ANOVA, *p*<0.0001; Tukey posthoc test: Ctrl vs. Aβ *p*<0.0001; Aβ vs. Aβ+SPL *p*<0.0001; Aβ vs. Aβ+YPL *p*<0.001; Ctrl vs. Aβ+SPL *p*<0.0001; Ctrl vs. Aβ+YPL *p*<0.0001, *n*=15). (AFU: Absolute Fluorescence Units). **C** Exemplary images of GFAP signals after treatments. **D**, **E** CellRox^®^ immunoreactivity significantly increases after Aβ treatment, indicating higher ROS levels. The increase in ROS induced by Aβ is significantly reduced in SPL and YPL-treated astrocytes (one-way ANOVA, *p*=0.0068; Tukey posthoc test: Ctrl vs. H_2_O_2_ *p*=0.0015; Ctrl vs. Aβ *p*=0.0254; Aβ vs. Aβ+SPL *p*<0.0001; Aβ vs. Aβ+YPL *p*<0.001; Ctrl vs. Aβ+SPL *p*<0.0001; Ctrl vs. Aβ+YPL *p*<0.0001, *n*=10). **E** Exemplary images of CellRox^®^ fluorescence after treatments. **C**, **E** Scale bar = 30 μm

To understand whether the effects in PTAFR levels seen by SPL and YPL application translated into changes in the general activation of astrocytes, we again analyzed GFAP expression using Western blotting (Fig. 4A) and quantified GFAP immunofluorescence (Fig. 4B,C). The results show that treatment with both SPL and YPL can prevent an increase in GFAP levels (Fig. 4A-C). Two housekeeping proteins (ACTIN and VINCULIN) were used, and the conditions normalized to their respective controls. While a complete block of GFAP increase was measured by protein biochemistry, the immunofluorescence results show only partial prevention of the Aβ-induced astrocyte activation measured by GFAP signal intensity. However, GFAP levels were still significantly lower after Aβ exposure with treatment with PLs (Fig. 4B). In addition, we could observe that Aβ-induced astrocyte ROS production was prevented by treatment with SPL and YPL (Fig. 4D,E). Treatment of cells with SPL and YPL alone, in the absence of Aβ, showed no effect on general astrocyte GFAP levels but also lowered ROS levels in healthy cells without Aβ exposure (Supplementary Fig. S2A-E). Besides, we analyzed the levels of the inflammatory cytokine IL-6 generated in astrocytes (Supplementary Fig. S3). The accumulation of cytokines characterizes neuroinflammation in AD. Among these IL-1β, IL-6, TNF-α, and TGF-β feature prominently. Their release can progress AD pathology and activate other glial cells [31] and is triggered by the presence of certain Aβ species, such as Aβ_1-42_ [32, 33]. Increased levels of IL-6 activate astrocytes, in return, will produce further pro-inflammatory cytokines [34], possibly by activating NF-κB [35, 36] driving the brain to a neuroinflammatory state. Our results show that SPL treatment prevents an Aβ-induced increase in *il-6* mRNA expression (Supplementary Fig. S3).

Overall, the data confirms that SPL and YPL are potent inhibitors of the PTAFR pathway. Interestingly, their biological activity results in the attenuation of astrocyte activation, indicating that PAF signaling is not only contributing but may be a key driver of Aβ-induced astrocyte activation.

Next, we investigated the impact of PL-attenuated PTAFR signaling on AD pathology. A key pathology of AD is Aβ aggregation, the loss of synapses, and neuronal death (neurodegeneration), which are linked to the clinical phenotype of AD. Therefore, we assessed these features *in vitro* using primary neuronal cells. We cultivated these rat hippocampal neurons for 14 days, which allowed them to mature into adult neurons [37]. Subsequently, we exposed those neurons to the secretome of astrocytes. To that end, we collected medium from astrocytes exposed to the following conditions: Untreated controls, astrocytes treated with Aβ, astrocytes treated with SPLs and YPLs, and astrocytes treated with SPLs and YPLs followed by Aβ.

Based on the results described above, we hypothesized that media of astrocytes that were activated by Aβ are rich in inflammatory cytokines such as IL-6 and ROS, while the presence of PLs should result in lower cytokine and ROS levels due to the observed reduction in astrocyte activation. From several studies, it is known that Aβ induces the production of ROS, NO, and cytokines such as IL-1β, IL-6, TNF-α, and IFN-α in astrocytes [38, 39], thereby affecting neuronal cell health. This was linked to the activation of signaling pathways, including the Janus kinase (JAK)/STAT3, the calcium/calcineurin (CN)/nuclear factor of activated T-cells (NFAT), NF-κB, and the MAPK pathway [40]. However, so far, the contribution of the PTAFR has not been investigated.

It has also been shown that cytokines released by astrocytes may influence the accumulation and clearance of Aβ, potentially contributing to its buildup and toxicity in the brain. Besides, factors released by astrocytes can impact synaptic pruning, leading to the loss of synapses and cognitive impairment [41]. For example, a correlation was found between the reduced synapse density and an increase in GFAP-positive astrocytes in AD patients, supporting a role for astrocytes in synapse elimination [42]. Primary rat hippocampal neurons exposed to Aβ-treated astrocytes have reduced expression of synaptic proteins [43].

**Fig. 5:**
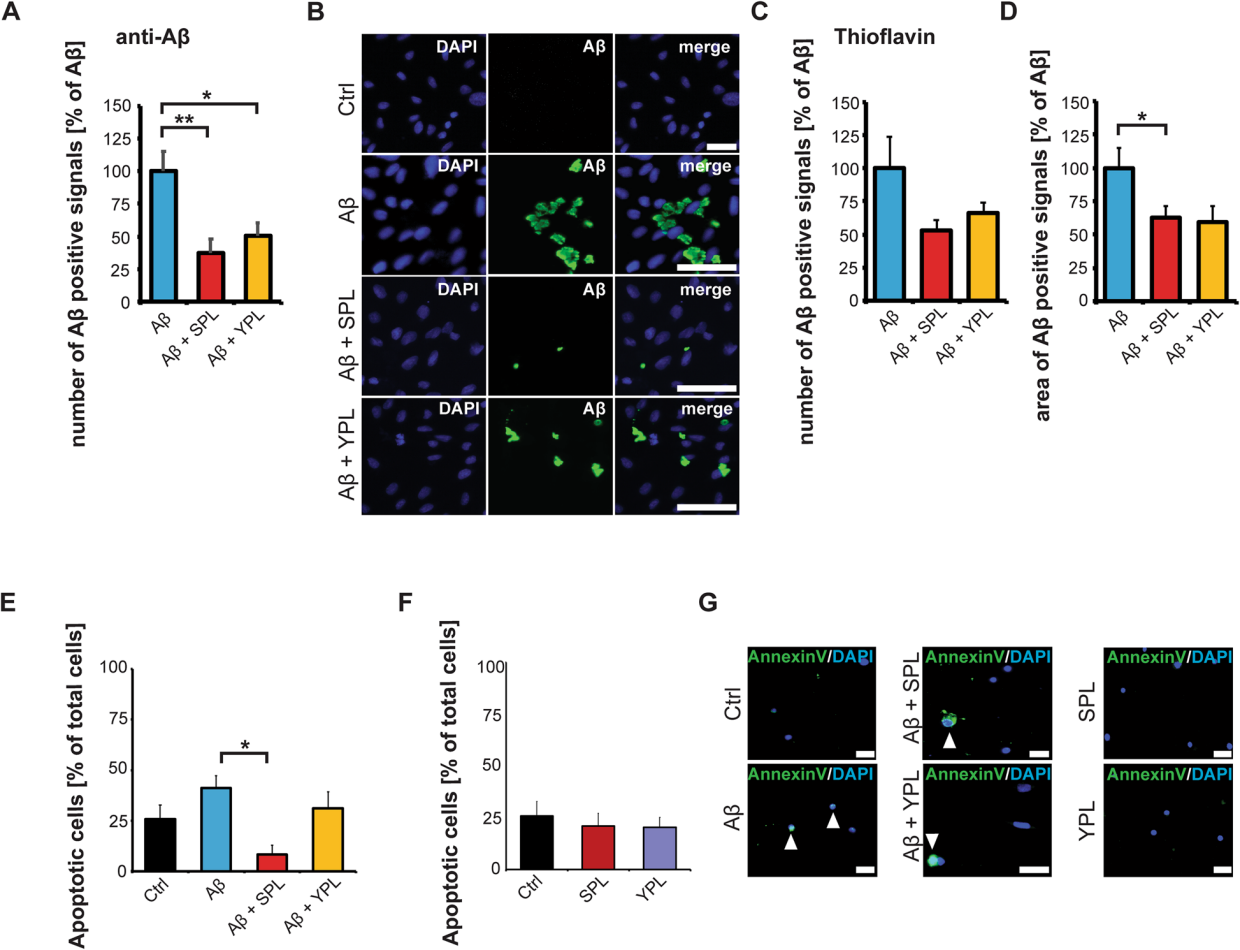
**A-C)** DI TNC1 cells were treated with 1 µM Aβ peptide for 24 h, and Aβ with SPL and YPL, and compared to untreated controls. **A**, **B** Immunolabeling of Aβ peptides using anti-Aβ antibodies reveals a reduction in the number of Aβ positive signals after treatment with PLs (one-way ANOVA, *p*=0.0037; Tukey post-hoc test: Aβ vs. Aβ+SPL *p*=0.044; Aβ vs. Aβ+YPL *p*=0.0247, *n*=10). **B** Exemplary images of Aβ signals after treatments. **C**, **D** Thioflavin labeling of Aβ shows no significant reduction in the number of Aβ aggregates (**C**) (one-way ANOVA), but a reduction in the Aβ aggregate size after SPL treatment (one-way ANOVA, *p*= 0.0141; Tukey post-hoc test: Aβ vs. Aβ+SPL *p*=0.0138, *n*=10) A reduction in Aβ aggregate size was also observed after YPL treatment, although not statistically significant (Tukey post-hoc test: Aβ vs. Aβ+YPL *p*= 0.0568, *n*=10) (**D**). **E**-**G** Primary hippocampal neurons were grown for 11 days and exposed to the media (secretomes) of astrocytes (untreated, treated with Aβ, treated with SPL or YPL plus Aβ, and treated with SPL or YPL only). After 4 days, the percentage of apoptotic cells per total cells in the culture was calculated. **E** The secretome of astrocytes exposed to SPL and Aβ caused significantly less effect on cell health compared to the secretome of astrocytes exposed to Aβ-only treatment (one-way ANOVA, *p*=0.012; Tukey post-hoc test: Aβ vs. Aβ+SPL *p*=0.00682, *n*=6-8 optic fields of view). **F** No effect on cell health was observed after exposure of neurons to the secretomes of astrocytes treated with SPL or YPL only. **G** Exemplary images of Annexin V FITC signals after treatments. White arrows indicate apoptotic cells. **B**, **G** Scale bar = 30 μm

In the first step, we analyzed Aβ clearance in the astrocyte cultures treated with Aβ and Aβ plus PLs (Fig. 5A-D). Using an anti-Aβ antibody, we detected a significant decrease of extracellular total Aβ in cultures treated with SPL and YPL (Fig. 5A,B). Additionally, while the number of Aβ aggregates was not changed (Fig. 5C), the Aβ aggregates measured by thioflavin had a significantly reduced size in cultures exposed to SPL (Fig. 5D). The supernatant of these cells was then transferred to primary mature hippocampal neurons.

We then harvested the medium containing the secretome of the treated astrocytes and exposed 11-day-old primary neurons to the astrocyte medium for 4 days. We measured the density of excitatory synapses using the synaptic marker SHANK3 (Supplementary Fig. S4). We evaluated the number of SHANK3 immunoreactive puncta along a MAP2-labeled dendrite. Our results show that the overall morphology of neurons, measured by MAP-2 labeling and counting of the mean number of primary, secondary, and tertiary dendrites, is not affected by the treatment of neurons with any of the secretomes of astrocytes exposed to Aβ, SPL, YPL, and SPL or YPL plus Aβ (Supplementary Fig. S4A-C). The average number of SHANK3 immunoreactive puncta along primary and secondary dendrites was also not significantly altered after treatment of neurons with the secretomes of astrocytes exposed to Aβ, SPL, YPL, and SPL or YPL plus Aβ (Supplementary Fig. S4D-F).

We also assessed the cell health of neurons by fluorescent Annexin V labeling (Fig. 5E-G). Our results show a trend towards an increase in apoptotic cells after exposure to the secretome of astrocytes treated with Aβ (Fig. 5E). A significant reduction of the number of apoptotic cells in the neuronal culture treated with SPL and Aβ was seen compared to neuronal cultures exposed to the secretome of astrocytes treated with Aβ only (Fig. 5E, G). Treatment with YPL showed no significant effect. However, treatment with YPL also did not result in a significant reduction in Aβ aggregation size in the astrocyte culture (Fig. 5D). The secretome of astrocytes treated with SPL and YPL in the absence of Aβ did not affect neuronal cell health (Fig. 5F, G). Thus, SPL may prevent neurodegenerative effects caused by chronic astrocyte activation by reducing PTAFR-mediated astrocyte activation.

## Discussion

Neuroinflammation plays a pivotal role in the progression of Alzheimer's disease. The activation of immune cells in the brain, particularly microglia and astrocytes, is a key factor driving neuroinflammation. This process is associated with the release of pro-inflammatory cytokines, such as IL-1β, TNF-α, and IL-6, leading to chronic inflammation and neuronal damage [44]. Several studies have highlighted the involvement of various signaling pathways and molecules in neuroinflammation within AD [45, 46]. Therefore, astrocytes have emerged as key contributors to the disease process. In AD, astrocytes undergo functional and morphological changes, and release pro-inflammatory cytokines, which exacerbate the inflammatory response in the brain. Research has shed light on various mechanisms through which astrocytes impact AD. Notably, the dysregulation of calcium signaling, altered lipid metabolism, and impaired Aβ clearance by astrocytes have been identified as significant contributors to disease progression [47, 48].

In this study, we report a key role of PTAFR-induced signaling for the pathomechanisms of chronically active astrocytes in AD. This pathway is activated by PAF and can be modulated by the presence of polar lipids. There are two main biosynthetic pathways of PAF in the brain. These include the remodeling and *de novo* pathways [16]. In addition, a third pathway exists under increased oxidative stress, known as the oxidative fragmentation pathway. The enzymatic remodeling pathway is thought to be associated with the biosynthesis of PAF in pro-inflammatory cells such as astrocytes. PAF subsequently binds to the PTAFR, initiating intracellular signaling cascades.

Our data show the presence of the PTAFR signaling pathway in astrocytes and that Aβ_1-42_ triggers PTAFR signaling. The levels of PTFAR significantly increase in astrocytes after exposure to Aβ, similar to levels reached by the induction of inflammatory signaling using the known and widely used stimulant LPS. Moreover, Aβ, as LPS, leads to an overall change in astrocyte reactivity. Notably, the application of PLs known to be PTAFR signaling inhibitors prevented increased PTAFR expression and overall astrocyte activation.

It has been shown that the Aβ peptide may activate astrocytes through its similarity with pro-inflammatory cytokines and anti-microbial peptides [49, 50]. For example, RAGE can bind Aβ. Activation of RAGE by Aβ leads to further activation of downstream signaling pathways such as p38 MAPK and NF-κB [4, 5]. Notably, the PTAFR signaling pathway also intersects with the NF-κB pathway. Thus, here, Aβ via RAGE and the PTAFR have a common downstream target (NF-κB). Prevention of NF-κB activation by blocking the PTAFR may thereby influence the ability of Aβ to activate NF-κB via RAGE.

Blocking RAGE signaling was shown to prevent toxicity mediated by oligomeric and aggregate forms of Aβ, but not fibrillar Aβ [51], suggesting that different Aβ conformations interact with distinct RAGE sites. However, RAGE is also able to bind Aβ fibrils [52]. Our method for preparing Aβ should result in mostly, but not entirely, monomeric Aβ. However, Aβ aggregates as soon as it is in the cell culture medium and during the relatively long exposure period with Aβ (24 h) used in this study, aggregation of Aβ occurs (evidenced by the presence of Thioflavin positive signals). Unfortunately, the approach makes it impossible to determine what form of Aβ activates the astrocytes, and whether PTAFR inhibition by PLs is specific for this type of activation. Besides, the production of pro-inflammatory molecules such as cytokines, and potentially PAF, can further activate astrocytes, leading to production of cytokines [52]. PTAFR signaling may also intersect with, or be, a signalling pathway not directly involved with the cellular process activated by Aβ, but secondarily by the Aβ induced release of cytokines.

Understanding the intricate involvement of neuroinflammation in AD opens avenues for potential therapeutic interventions targeting neuroinflammation to alleviate disease progression. In the past, multiple studies showed improved cognitive performance in both middle-aged and older individuals who regularly consumed fish [53, 54].

Omega-3 fatty acids, particularly eicosapentaenoic acid (EPA) and docosahexaenoic acid (DHA) have been investigated for their potential role in the prevention of AD. Cross-sectional studies indicated a lower risk of dementia-related diseases, such as AD, through DHA intake, which is typically directly related to fish consumption [55, 56]. Studies suggest that omega-3 fatty acids possess neuroprotective properties, influencing various aspects of AD pathophysiology. DHA, in particular, is believed to help reduce inflammation, oxidative stress, and the aggregation of Aβ [57]. Clinical and epidemiological evidence has indicated that higher consumption of omega-3 fatty acids through fish or supplements is associated with a reduced risk of cognitive decline and AD. However, the outcomes from intervention studies exploring the direct impact of omega-3 supplementation on AD prevention have been somewhat inconclusive [58–60].

Dairy products such as milk and yogurt are rich sources of polar lipids that possess significant health benefits, such as improvement of cognitive health, antiplatelet and anti-inflammatory properties [29, 61, 62]. Clinical studies have demonstrated that supplementation with polar lipids derived from dairy improve cognitive functioning and neurodevelopmental outcomes [27, 63]. Fatty acids derived from milk, yogurt and other dairy products have established protective effects of polar lipids and other fatty acid substances against inflammatory pathways [29, 64–66].

Several fish, such as salmon, are excellent sources of marine PLs with strong preventative bioactivities against PTAFR as competitive inhibitors of the PAF ligand. The Irish organic farmed salmon used in this study is rich in ω3 PUFA, which included the most plentiful ω3 fatty acids EPA and DHA (Table. 1). The presence of these ω3 fatty acids in salmon coupled with anti-PTAFR polar lipids appear to exert the anti-inflammatory effects observed *in vitro*. Indeed, we could measure that treating astrocytes with SPL before activation with Aβ_1-42_ results in fewer effects on cell health, and thus likely neurodegeneration, in our neuronal model. It is important to mention that these neurons were not directly exposed to Aβ_1-42._ Therefore, we only measure the effects of the over-activation of astrocytes on neurons through their release of molecules (the secretome), likely consisting of relevant factors such as inflammatory cytokines or ROS. Therefore, we may not have observed the often-reported effects of direct exposure to Aβ_1-42,_ such as a reduction in synapse numbers [67]. In addition, it is important to highlight that the astrocytes in our study were activated for 48 hours in total. Thus, while their activation may initially be a protective mechanism, the experimental design here leads to an extended and chronic accumulation of released molecules in the astrocyte medium. This may be more reflective of situations of chronic neuroinflammatory signaling.

The interplay of different cell types with Aβ in AD is more complex than modelled here, with direct effects of Aβ_1-42_ on neurons and also microglia, plus indirect effects of their secretomes. For example, IL-6 released by astrocytes may activate microglia that in turn release a plethora of cytokines acting on neurons [68, 69]. However, the study reveals that the PTAFR receptor pathway activates astrocytes and its attenuation may protect brain cells from the adverse effects mediated by the secretome of chronically activated astrocytes in AD. The study also proposes one mechanism by which PLs may exert their reported protective effects. More research is needed to explore this mechanism as a new potential drug target in AD.

Treatment with YPL seemed less effective on neurons despite astrocytes reacting to both SPL and YPL similarly. A comparison of the lipid profiles of each PL extract reveals that YPL extracts have different PL compositions with less DHA and EPA than SPL. In our studies, we have analyzed selected end-points. It is possible that YPL and SPL extracts are both effective, but the temporal dynamics may be different. The treatments may both reduce astrocyte activation, but at different times, which will influence the accumulation of molecules in the medium, and thus, may generate different effects on neurons. For example, if SPL is more potent in reducing astrocyte activation, the threshold of released molecules to act detrimentally on neurons may not have been reached in contrast to the less effective YPLs that deactivate astrocytes, but potentially later, resulting in higher levels of released molecules.

## Conclusions

Here, we show that the decrease in astrocyte activation is at least partly based on the inhibitory activity PLs exert on PTAFR signaling. Therefore, increased dietary levels of PLs may reduce the activation of astrocytes, thereby decreasing cytokine release within the brain and, ultimately, neuroinflammation. Our results highlight a novel underlying mechanism and why consuming PL-rich foods such as fish and dairy may reduce the risk of developing dementia and associated disorders. Moreover, enriching foods with PLs to render them functional, PLs' use as nutraceuticals, and the use of different sources with optimal PL profiles should be further explored as preventive and therapeutic strategies in AD. Overall, identifying the PTAFR as a critical cellular signaling component outside the commonly associated platelet activation mediated by PTAFR allows exploring PTAFR signaling as a drug target in AD.

### Supplementary Information

**Supplementary Material 1.** 

## Data Availability

The data that support the findings of this study are available from the corresponding author upon reasonable request.

## Abbreviations

AD: Alzheimer’s disease
DHA: Docosahexaenoic acid
EPA: Eicosapentaenoic acid
DPA: Docosapentaenoic acid
HMBS: Hydroxymethylbilane synthase
LC-MS: Liquid chromatography–mass spectrometry
MAP2: Microtubule associated protein 2
Shank3: SH3 and multiple ankyrin repeat domains 3
MUFA: Monounsaturated fatty acids
n-6: Omega-6
n-3: Omega-3
ND: Non-detectable
PL: Polar lipids
PTAFR: Platelet-activating factor receptor
PUFA: Polyunsaturated fatty acids
qRT-PCR: Quantitative reverse-transcriptase polymerase chain reaction
ROS: Reactive oxygen species
SPL: Salmon polar lipids
SFA: Saturated fatty acids
WB: Western blot
YPL: Yogurt polar lipids
