# Supplementary material for: Polar lipids modify Alzheimer’s Disease pathology by reducing astrocyte pro-inflammatory signaling through platelet-activating factor receptor (PTAFR) modulation
Source: Lipids Health Dis. 2024 Apr 20;23:113. doi: 10.1186/s12944-024-02106-z (PMC11031880; doi:10.1186/s12944-024-02106-z)
Supplement: Supplementary file 1 — Supplementary Material 1. [file 12944_2024_2106_MOESM1_ESM.docx]

S U P P L E M E N T A R Y M A T E R I A L

Polar lipids modify Alzheimer's Disease pathology by reducing astrocyte pro-inflammatory signaling through platelet-activating factor receptor (PTAFR) modulation

Sakshi Hans | Janelle E. Stanton | Ann Katrin Sauer | Katie Shiels | Sushanta Kumar Saha | Ronan Lordan | Alexandros Tsoupras | Ioannis Zabetakis | Andreas M. Grabrucker

**SUPPLEMENTARY FIGURES**

Figure S1


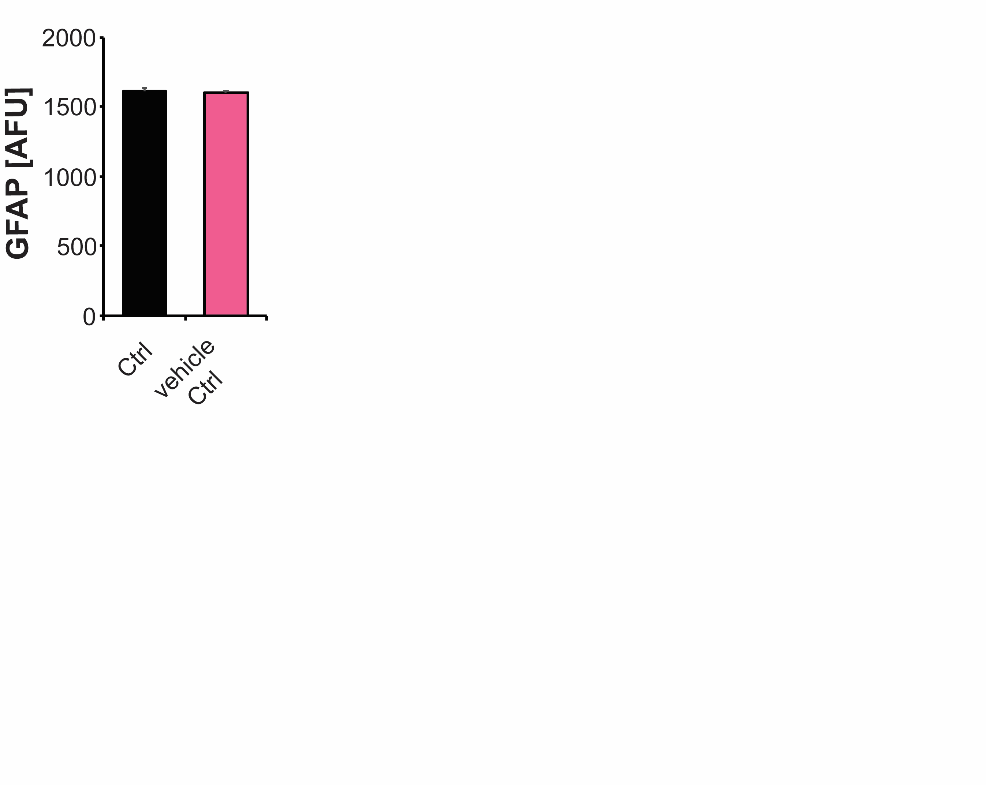


**Figure S1:** DI TNC1 cells were treated with vehicle control for 1 µM Aβ peptide for 24 h and compared to untreated controls. Immunocytochemistry (ICC) labeling of GFAP shows no significant difference between vehicle and untreated controls (AFU: Absolute Fluorescence Units).

Figure S2


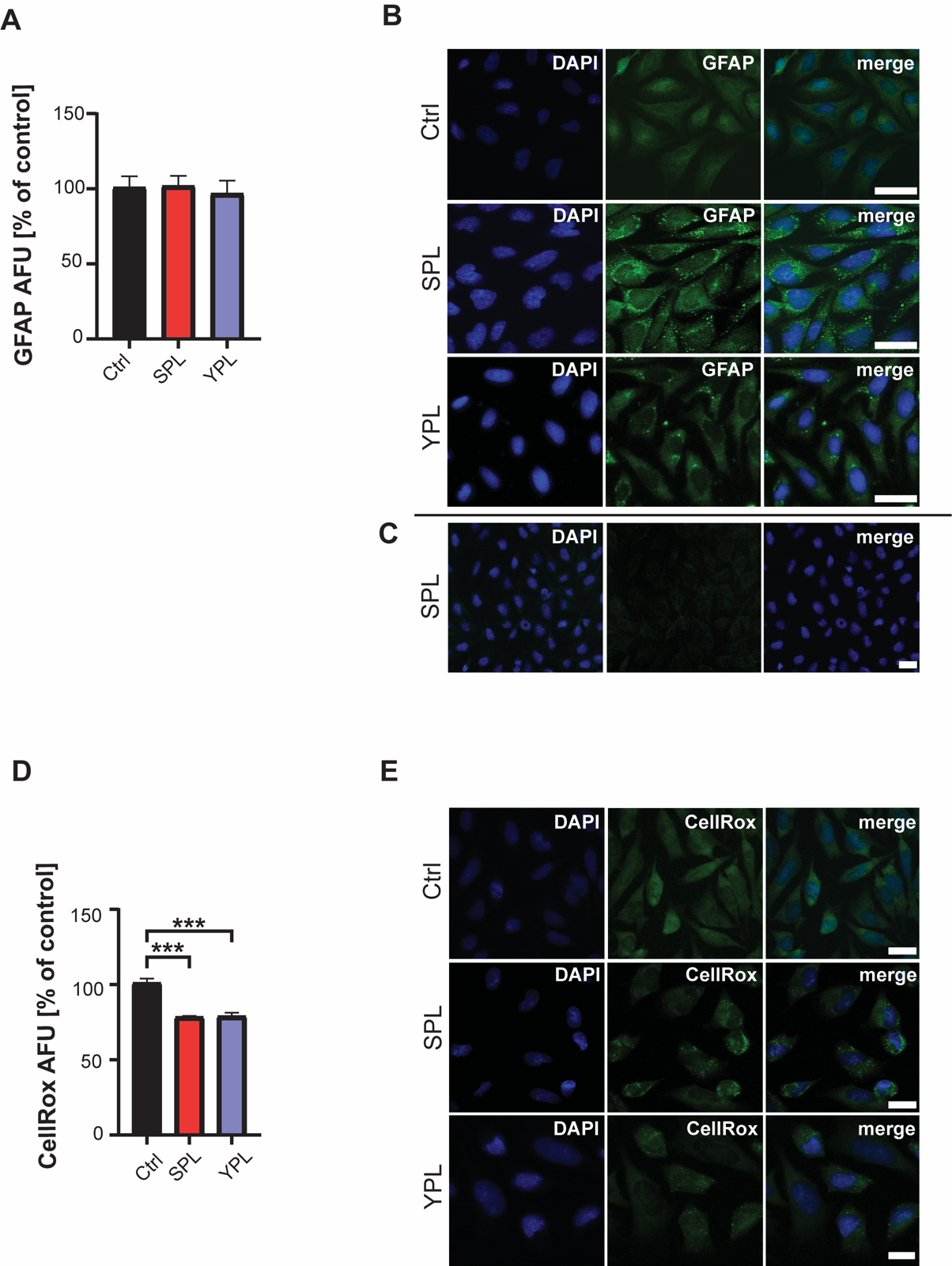


**Figure S2: A-E)** DI TNC1 cells were treated with 1 µM Aβ peptide for 24 h, and Aβ with SPL and YPL compared to untreated controls and positive controls. **A,B)** Immunocytochemistry (ICC) labeling shows no effects of SPL or YPL alone on GFAP levels (one-way ANOVA p=0.1744, n=10) **B)** Exemplary images of GFAP signals after treatments. SPL and YPL-treated cells show vesicular structures with higher GFAP fluorescence. **C)** Staining without primary antibody was performed to confirm that these signals are GFAP positive and not background caused by lipids in vesicles. No signals were detected here, confirming that the vesicles observed after SPL and YPL treatment are indeed GFAP positive. **D,E)** CellRox^®^ signals are significantly decreased after treatment with SPL or YPL alone, revealing an anti-oxidative effect of PLs also on healthy cells (one-way ANOVA, p<0.0001; Tukey post-hoc test: Aβ vs. Aβ+SPL p<0.0001; Aβ vs. Aβ+YPL p<0.0001, n=10). **D)** Exemplary images of CellRox^®^ fluorescence after treatments. **B,C,E)** Scale bar = 15 μm.

Figure S3

**
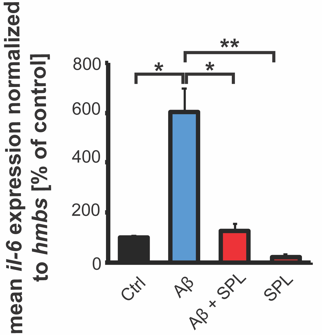
**

**Figure S3:** DI TNC1 cells were treated with 1 µM Aβ peptide for 24 h, and Aβ with SPL and YPL, and compared to untreated controls and positive controls. Detection of *Il-6* in astrocytes on mRNA level using qRT-PCR**.** Aβ treatment triggers an increase in *il-6* expression that is normalized by SPL application (one-way ANOVA, p=0.0104; Tukey posthoc test: Ctrl vs. Aβ p=0.0231; Aβ vs. Aβ+SPL p=0.0278; Aβ vs. SPL p=0.0093, n=3).

Figure S4


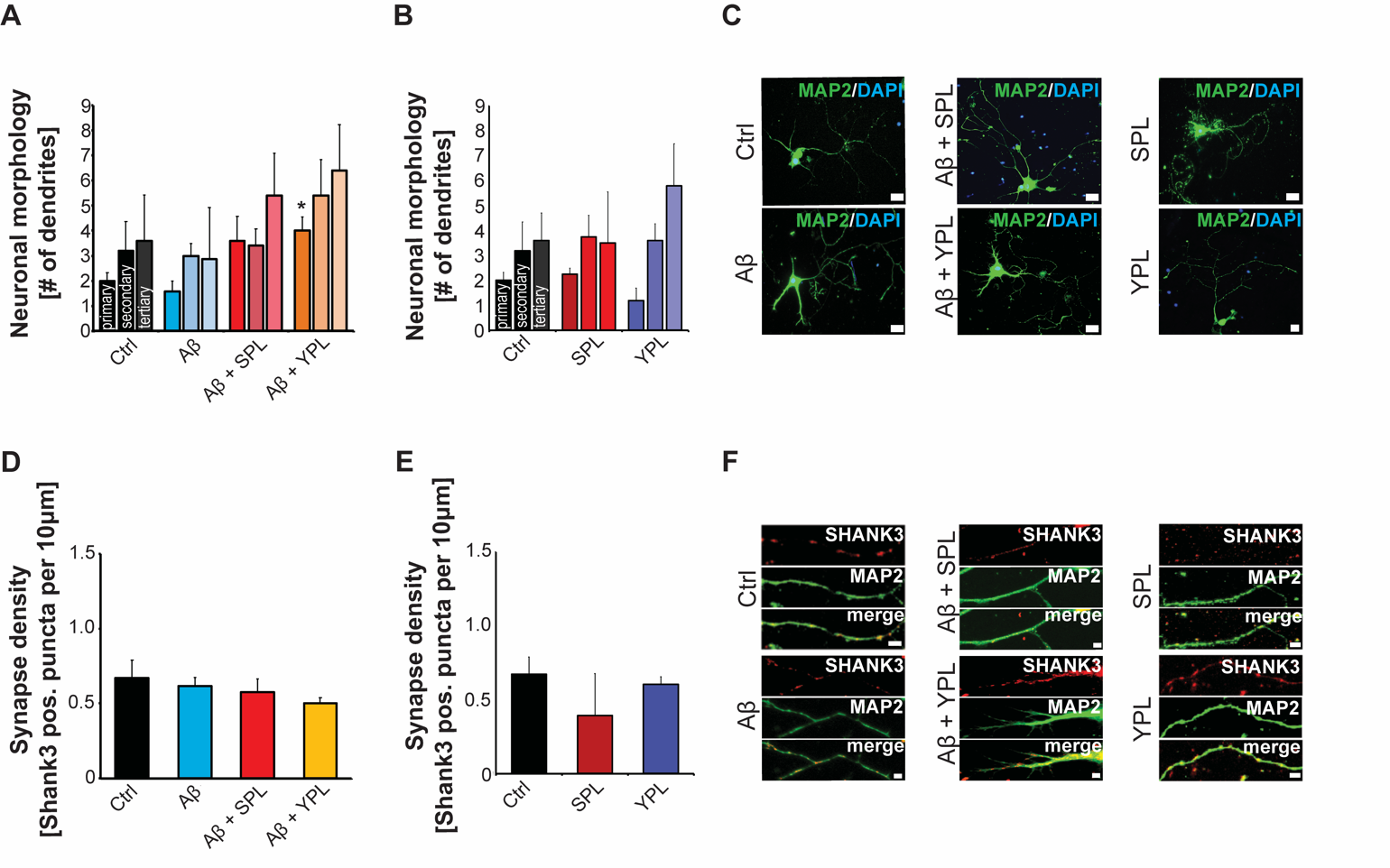


**Figure S4:** Primary hippocampal neurons were grown for 14 days and exposed to the media (secretomes) of astrocytes (untreated, treated with Aβ, treated with SPL or YPL plus Aβ, and treated with SPL or YPL only). **A,B)** Dendritic branching measured by the average number of primary, secondary, and tertiary dendrites per cell was not affected by any secretome except for the number of primary dendrites being significantly higher in neurons exposed to medium of astrocytes treated with YPL plus Aβ compared to Aβ (one-way ANOVA, p=0.0078; Post-hoc test: Aβ vs. Aβ+YPL_primary_ p=0.0337, n=5-7). **C)** Exemplary images of MAP2 and DAPI fluorescence after treatments. Scale bar = 30 μm. **D,E)** The average number of SHANK3 immunoreactive signals along a primary or secondary dendrite per neuron was unaffected by any secretome **F)** Exemplary images of MAP2 and SHANK3 fluorescence after treatments. Scale bar = 5 μm.
